# Agricultural Genomics: Commercial Applications Bring Increased Basic Research Power

**DOI:** 10.1371/journal.pgen.1005621

**Published:** 2015-11-05

**Authors:** Jared E. Decker

**Affiliations:** 1 Division of Animal Sciences, University of Missouri, Columbia, Missouri, United States of America; 2 Informatics Institute, University of Missouri, Columbia, Missouri, United States of America; Georgia Institute of Technology, UNITED STATES

Homologous recombination has been a focus of basic research for over a hundred years [1–3]. The advent of genomics has allowed fine-scale analyses of recombination, including genome-wide analysis of global recombination rate [4–6] and hotspot usage (the proportion of recombination that occurs at hotspots) [4,6]. Recently, *PLOS Genetics* published the work of Ma et al. [7] describing a genome-wide association study of global recombination rate in Holstein cattle using 3,224 males and 53,125 females and hotspot usage using 1,772 males and 12,756 females. The recombination rate and hotspot usage phenotypes were estimated using 223,364 samples that belonged to 185,917 three-generation families, which were extracted from the larger pedigree that contained over half a million Holstein cattle when the research was conducted.

## Why Genotype Half a Million Cattle?

**Fig 1:**
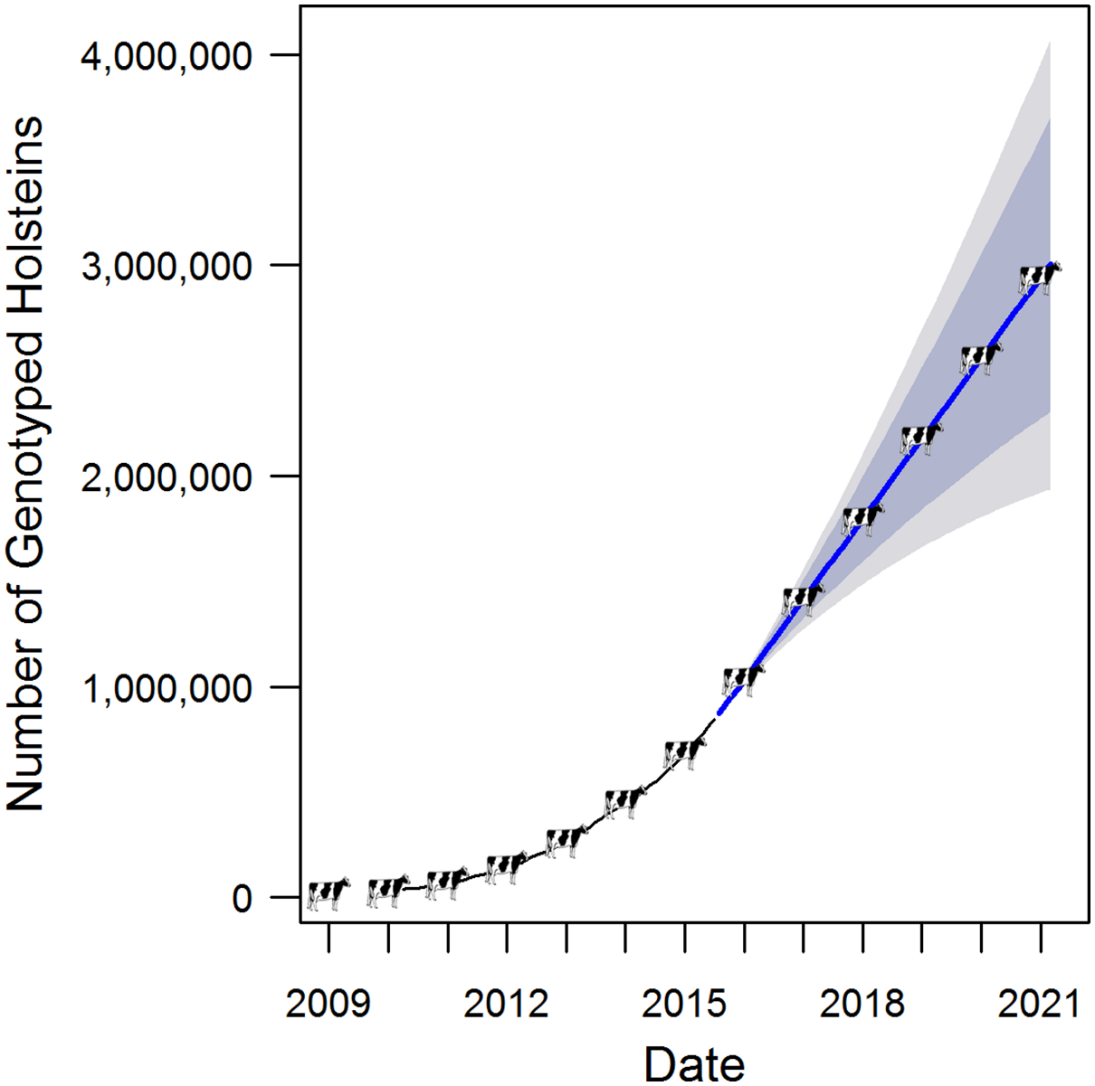
Number of Holsteins genotyped since 2009. The cumulative numbers of Holsteins genotyped from January 1, 2009 to July 1, 2015 are plotted. Cumulative genotype counts from April 2010 to July 2015 were used in a time series ARIMA model (with 1st order autocorrelation, 2 degrees of differencing, and 4th order moving averages) to predict the number of genotyped animals till March 2021. Dark grey shading corresponds to 80% confidence interval, and light gray corresponds to 95% confidence interval. Cumulative number of animals genotyped was downloaded from the Council on Dairy Cattle Breeding website (https://www.cdcb.us/Genotype/cur_density.html). Data and R code use to generate the plot are available at https://missouri.box.com/Fig1-HolsteinPredictions.

In 1997 [8] and 2001 [9], researchers proposed methods to predict the genetic merit (i.e., their value as parents of the next generation) of organisms using dense genotype data; we now refer to these methods as genomic selection or, perhaps more accurately, as genomic prediction. Genomic prediction, whether using the estimated effects of variants merged with independently estimated merits based on phenotype and pedigree (multi-step) or by supplementing the pedigree-based expected relationships (average extent of allele sharing based upon pedigree relationship) with realized relationships estimated from genotype data (single-step), predicts the expected performance of an individual’s progeny relative to the population average using that individual’s genotypes at thousands of DNA variants. When genomic prediction was first proposed, there was not an affordable technology to genotype a sufficient number of variants in a large number of individuals. But, in 2008, a commercial single nucleotide polymorphism (SNP) array for cattle was released [10] and, in 2009, genomic prediction was implemented in United States Holstein dairy cattle. Since that time, genomic prediction has been implemented in many other livestock and crop populations. Genomic prediction solves the main weakness of traditional pedigree-based genetic merit predictions, namely that estimates are imprecise for young animals with little or no data on their progeny. By tracking the inheritance of segments of chromosomes, either through multi-step or single-step methods, genomic predictions provide the same amount of information as five to 30 progeny records (the number varies by breed and trait) [11]. With genomic prediction, the lack of data and predictive reliability for young animals is alleviated and the rate of genetic progress has increased dramatically. As demonstrated by Ma et al. [7], these genomic data sets not only have commercial applications to improve agriculture production but can also be used to answer basic biological questions. In 2009, The Bovine Genome Sequencing Consortium stated, “The cattle genome and associated resources will facilitate the identification of novel functions and regulatory systems of general importance in mammals…” [12]; the work of Ma et al. [7] is one of many fulfillments of that prediction. Because of the economic impact of genomic prediction, genomic data is being generated in agricultural species at an unprecedented rate. A time series analysis predicts that there will be over one million Holstein cattle genotyped by the end of 2015, and, in the next six years, that number will likely triple (Fig 1). In many cases, these genotype data are not publically available due to their commercial value, but, typically, breed associations have been willing to execute material transfer agreements with academic scientists for research complimentary or peripheral to their commercial objectives. Notably, after initial development of the genomic prediction, the genotyping of these animals is paid for by farmers and incurs little cost to the research community. In addition to SNP array data, the 1,000 Bull Genomes Project has processed whole-genome sequences for over 1,577 cattle, and many individual laboratories have resequenced hundreds of additional animals that are not yet part of the consortium data set. Combining this research-funded genomic sequence data with commercially-funded genotype array data will only increase the power to answer basic biology questions with agriculturally important species.

## Recombination Insights

With tens of thousands of cattle with recombination phenotypes, Ma et al. [7] confirmed associations between not only *PRDM9* paralogs, *CPLX1*, *REC8*, and recombination rate but also showed increased power to detect novel associations, such as *NEK9*, *REC114*, *MSH4*, *SMC3*, and *CEP55*. As a resource to the bovine genomics community, the authors have made their recombination maps and high-quality crossover data publicly available [7]. Furthermore, the researchers show that, unlike sex differences observed in humans and mice, bulls (males) have more recombination than cows (females), perhaps due to stronger selection intensity on artificial insemination sires compared with females. Further, the authors provide evidence that the male recombination rate may have decreased in the last 40 years. Comparative studies with beef cattle or other livestock species may elucidate the demographic forces behind these recombination sex differences and trends over time.
